# Biotechnological Approach Based on Selected *Saccharomyces cerevisiae* Starters for Reducing the Use of Sulfur Dioxide in Wine

**DOI:** 10.3390/microorganisms8050738

**Published:** 2020-05-15

**Authors:** Angela Capece, Rocchina Pietrafesa, Gabriella Siesto, Patrizia Romano

**Affiliations:** Scuola di Scienze Agrarie, Forestali, Alimentari ed Ambientali, Università degli Studi della Basilicata, 85100 Potenza, Italy; angela.capece@unibas.it (A.C.); rocchina.pietrafesa@unibas.it (R.P.); pot2930@gmail.com (P.R.)

**Keywords:** *Saccharomyces cerevisiae*, sulfur dioxide (SO_2_), mixed starter, NO-SO_2_ added wine, polyphenols, antioxidant activity, strain dominance

## Abstract

Sulfites are considered the main additives in winemaking for their antimicrobial, antioxidant and anti-oxidasic activities. The current concern about the potential negative effects of sulfur dioxide (SO_2_) on consumer health has focused the interest on replacing or reducing SO_2_ use. Our work aims to develop a strategy based on the use of selected starter culture, able to perform wine fermentation without SO_2_ addition. Four selected *Saccharomyces cerevisiae* indigenous strains were tested as mixed starter cultures in laboratory scale fermentations. The starter culture, characterized by a similar percentage of dominance of both strains composing the mixed starter and able to produce a wine characterized by the best combination of chemical and aromatic characteristics, was chosen. This mixed culture was tested as a starter at pilot scale with and without SO_2_ addition, by using a higher inoculum level in the vinification without SO_2_. The selected starter confirmed higher dominance ability in vinification without SO_2_ addition than in SO_2_-added fermentation, demonstrating that sulfite addition is not a guarantee to reach an absolute dominance of starter culture on indigenous microflora. The proposed biotechnological tool allowed to produce good quality wines possessing also “functional properties”, as NO-SO_2_ added wines were characterized by high polyphenol content and antioxidant activity.

## 1. Introduction

The antioxidant and antimicrobial action of sulfur dioxide (SO_2_) is well known, being commonly used in many processed foods for hundreds of years [1], especially in low pH foods, such as grape must. The most important role of this compound lies in its antimicrobial action against acetic and lactic acid bacteria, and molds in order to prevent spoilage and to determine the microbiological stabilization of wines to enhance aging potential [2]. Furthermore, SO_2_ addition prior to the onset of alcoholic fermentation also exerts a selective antimicrobial activity against spoilage yeasts, by inhibiting their growth and promoting the rapid development of *Saccharomyces cerevisiae*. Due to these properties, winemakers considered sulfur dioxide indispensable, but the current concern about the potential negative effects of excessive amounts of this compound in wine on organoleptic quality and consumer health, has focused the interest on minimizing the amounts of SO_2_ in grape must and wine. The OIV (International Organization of Vine and Wine) has established limits for SO_2_ content in wines [3] in agreement with European Commission regulations. The total sulfur dioxide content cannot exceed 150 mg/L and 200 mg/L in red and white wines, respectively, whereas organic red and white wines cannot contain more than 100 mg/L and 150 mg/L [4].

Numerous studies report the intolerance or high sensitivity to sulfite additives with consequent expression of a wide range of adverse effects, such as allergic responses with increasing risk of asthmatic attacks, trouble breathing, skin rashes, and stomach pain [5,6,7,8]. As a consequence, in recent years, a negative perception has been created towards sulfites, resulting in a significant increase in consumers’ demand toward wines with low SO_2_ content [9] and the research is focused on looking for other preservatives and innovative technologies, harmless to health, in order to reduce SO_2_ content in wine. Recently, numerous alternatives have been proposed to replace the activity of SO_2_ by the use of chemical additives and physical treatments, aimed at the microbiological stability of wine [10].

Among suitable chemical alternatives to SO_2_, different antimicrobial compounds are added in musts and wines, according to EU and OIV legislation, such as dimethyl dicarbonate (DMDC), able to inhibit microorganisms by reacting irreversibly with the amino groups of alcohol dehydrogenase [11] and very effective against yeasts [12]. The lysozyme is reported to exert a destructive action against the cell walls of Gram-positive bacteria [13], whereas sorbic acid was effective to inhibit refermentation by *S. cerevisiae* in bottled sweet wines [14,15] and towards the growth of film-forming yeasts (*Candida* spp.) on the wine surface [1].

Additionally, additives, authorized in winemaking for other scopes, were tested for potential antimicrobial action against wine spoilage microorganisms, such as phenolic compounds, chitosan and ß-glucanases [16,17,18,19]. Other chemical additives, whose use is not yet permitted in the EU, were tested for potential action in preventing wine microbial spoilage, such as nisin [20], silver nanomaterials [21], hydroxytyrosol [22] and saturated short-chain fatty acids [23]. In addition, *S. cerevisiae* can fight other microorganisms by applying defensive strategies by different mechanisms, such as the secretion of AMPs and cell-to-cell contact [24].

The general aim of reducing the addition of chemical additives is better satisfied in the application of physical methods, such as high hydrostatic pressure, ultrasound, pulsed electric fields, ultraviolet irradiation and microwave, successfully used in the last few years for the microbiological stabilization of wine as alternative to the use of SO_2_. It should be highlighted that currently no physical or chemical treatment, although exhibiting a certain microbial inhibition, has to date shown to be able to replace the efficacy and the broad spectrum of antimicrobial action of SO_2_ [25]. These alternatives can only find a useful application as a complement of SO_2_, allowing its reduction in winemaking. However, it must be underlined that wine cannot be completely free of sulfites, because SO_2_ is naturally produced during the fermentation process by yeasts. Depending on the yeast strain and also on the composition of the grape must, yeasts produce variable quantities of SO_2_ (on average about 10 mg/L, but also up to 100 mg/L) [26].

In this context, the worldwide trend toward the reduction of preservatives has stimulated the research for new alternatives with microorganisms as a primary source of antimicrobial agents (biocontrol). The use of selected mixed cultures of yeast has been reported to potentially improve the control of unwanted or spoilage microorganisms, thanks to interaction mechanisms that can ensure the dominance of the inoculated starter [27]. In addition, the behaviour as biocontrol agent has been exhibited by different genera of yeasts against some pathogenic bacteria or affecting the growth of other yeasts [28]. Strains of *Saccharomyces* are able to produce specific proteins, killer toxins, that prevent the growth of spoilage yeasts and molds in winemaking conditions [29]. Other studies report the antimicrobial activity of *Metschnikowia pulcherrima* against postharvest pathogens, microscopic fungi and other yeast species [30], determined by the secretion of the protein pulcherrimin [31]. Antifungal activity against *Brettanomyces*/*Dekkera* yeasts was exhibited by a strain of *Pichia anomala*, which secreted a killer toxin [32], while a strain of *Tetrapisispora phaffii* produced a killer toxin against apiculate yeasts [33].

Our research was directed to the development of a biotechnological strategy based on the use of specific indigenous strains of *S. cerevisiae*, able to perform the fermentation of grape must without adding sulfite. Therefore, in this study, we tested different strains of *S. cerevisiae*, already characterized for enological traits in order to select a strain combination characterized by a high ability to dominate the fermentative process. Two strains, selected on the basis of the highest ability to dominate laboratory scale fermentations and to produce a wine characterized by the best combination of chemical and aromatic characteristics, were tested as mixed starter in pilot-scale vinification, performed in a cellar, with and without sulfite addition. The ability of mixed starter to dominate the natural microflora and the evaluation of aromatic characteristics of final wine in fermentation without sulfite addition were used as criteria to establish the suitability of a selected mixed starter to be proposed as a tool for the production of wine with limited sulfite addition.

## 2. Materials and Methods

### 2.1. Saccharomyces cerevisiae Strains

Four indigenous *S. cerevisiae* strains, RB3-7Sc2 (ND), TA4-10 (TA), M1-47 (M1) and SA7-13 (SA), belonging to the UNIBAS Yeast Collection (UBYC), University of Basilicata (Potenza, Italy), previously isolated from the spontaneous fermentation of grapes of different varieties, were analyzed in this study. The strains were identified as *S. cerevisiae* species by amplification of the 5.8 S-ITS region, followed by a restriction analysis [34], and the analysis of D1/D2 domain of 26S rDNA [35]. The four strains were typed by analysis of interdelta region with δ2/δ12 primer pair [36], following the procedure reported by Capece et al. [37]. Origin and oenological traits of analyzed strains are reported in Table 1. The strains were stored at −80 °C in cryovials with YPD medium (1% (*w*/*v*) yeast extract; 2% (*w*/*v*) peptone, 2% (*w*/*v*) glucose, Oxoid, Hampshire, UK) and glycerol as protective agent (25% *v*/*v*, Sigma, USA).

### 2.2. Fermentation Assays

#### 2.2.1. Laboratory-Scale Fermentations

**Figure 1:**
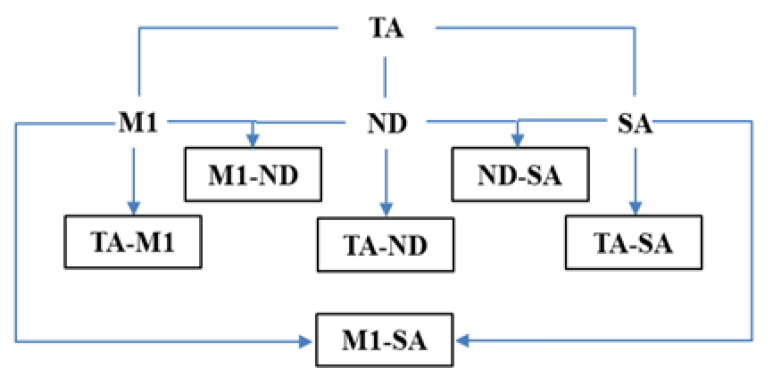
Scheme of lab-scale vinification trials. TA, M1, SA and ND = single fermentations using TA, M1, SA and ND starters, respectively; TA-M1 = co-fermentation with TA and M1 strains; M1-SA = co-fermentation with M1 and SA strains; ND-SA = co-fermentation with ND and SA strains; TA-ND = co-fermentation with TA and ND strains; TA-SA = co-fermentation with TA and SA strains; ND-M1 = co-fermentation with ND and M1 strains.

Duplicate fermentations were conducted in 500 mL of pasteurized “Aglianico del Vulture” grape must (pH 3.46; with an initial density of 1.101 g/L, equivalent to a sugar content of 223 g/L; YAN 236.02 mg/L). The absence of viable cells was checked by plate counting on Wallerstein Laboratory Nutrient Agar medium (WL, Oxoid, Hampshire, UK, [42]). The strains were tested as mixed starter cultures, in which each strain was inoculated in co-culture with each of the other strains (Figure 1), by adding, for each strain, 1 × 10^7^ cells/mL, to obtain 2 × 10^7^ cells/mL as total inoculum; as control, pure culture fermentations with each selected *S. cerevisiae* strain at a concentration of 2 × 10^7^ cells/mL were used.

In total, twenty fermentations (4 by single cultures + 6 mixed fermentations, 2 replicates) were carried out. The yeast cells were precultured in YPD at 26 °C for 24 h. After inoculation, the single and mixed fermentations were incubated at 26 °C without agitation. During the process, the fermentative course was monitored by measuring weight loss and sugar consumption by refractometric analysis. To evaluate the strain dominance level of starter cultures, samples were taken at specific stages of each laboratory fermentation, in particular at the middle (50% sugar consumption) and at the final phase of the process, and plated on WL medium.

From statistically representative plates, around 30 colonies (referable to *S. cerevisiae*) were randomly isolated from each fermentation sample and purified on YPD plates for analysis of inter-δ region, in order to compare the molecular profile of the isolates with those of the inoculated *S. cerevisiae* strains. Fermentations were assessed as completed when no further weight loss was recorded for three consecutive days. At the end of the fermentation process, experimental wines were refrigerated, transferred in sterile falcons and stored at 4 °C until analyses of conventional chemical parameters and aromatic compounds were carried out.

#### 2.2.2. Fermentations at Pilot-Scale

On the basis of strain dominance level detected at laboratory-scale fermentations, the mixed starter culture ND-SA was chosen to perform the fermentation at pilot scale in a winery located in Basilicata, Southern Italy. The two strains were refreshed on YPD plates and incubated at 26 °C for 24 h. A loopful of 24 h culture of each strain was inoculated in 500 mL of YPD broth and incubated at 26 °C for 24 h, in a rotary shaker at 180 rpm. The inoculum of each strain for cellar fermentation was produced by the BioFlo/CelliGen 110 (Eppendorf, Germany) bioreactor in a vessel containing 4.5 L of YPD liquid, as a growth medium, inoculated with 500 mL of the pre-inoculum of each strain. The growth parameters used were: controlled temperature at 26 °C; stirring at 400 rpm; oxygen at 4 vvm.

After overnight growth in the bioreactor, the level of cell proliferation was measured spectrophotometrically at 600 nm and a suitable amount of the culture broth of each strain was centrifuged at 4700 rpm. The recovered biomass was kept at 4 °C until the use. In the cellar, the two strains were inoculated as mixed culture, by using the same inoculation level reported for laboratory scale fermentation, in 370 L of “Aglianico del Vulture” grape must (the same used for the laboratory-scale fermentation). Two different fermentation modalities were performed in duplicate: (a) grape must with addition of 50 ppm of sulfur dioxide and inoculum of 1.4 × 10^6^ cells/mL of the mixed culture; (b) grape must without addition of sulfur dioxide and inoculum of 2.0 × 10^6^ cells/mL of the mixed culture. At the middle (50% of sugar consumption) and final step of the fermentation, samples from each vinification were plated on WL medium and 50 yeast colonies were isolated and identified by restriction analysis of amplified ITS region [34]. The colonies belonging to *S. cerevisiae* species were submitted to amplification of interdelta region with δ2/δ12 primer pair, in order to evaluate the dominance level of the starter culture [36,37].

### 2.3. Chemical Analysis

Conventional chemical parameters, such as total and volatile acidity, residual sugars, alcohol, pH were evaluated by Fourier Transfer Infrared WineScan instrument (FOSS, Hillerød, Denmark).

*Determination of SO_2_*—The free SO_2_ content was analytically determined by iodometric titration according to the Ripper method [43]. In this assay, the samples were acidified with H_2_SO_4_ (20%) and were titrated with a standard iodine solution (0.01N), using starch (1%, Oxoid, Hampshire, UK) as the indicator until the endpoint of titration (change of color to dark-blue). Total SO_2_ was determined by adjusting the sample pH to alkaline conditions (with NaOH 1M) and then incubating in the dark to release the bound fraction as free, which was determined as previously described. The content of SO_2_ (free or total) was determined on the basis of the consumed volume of I_2_ for titration of the sample.

*Determination of total polyphenols content of experimental wines—*The total polyphenols present in the experimental wines were measured spectrophotometrically after the reaction with Folin-Ciocalteu reagent (Sigma, USA), following the protocol described by Singleton et al. [44] and Stratil et al. [45]. The total volume of the reaction mixture was 10 mL. The absorbance was measured at 765 nm against blank. The concentration of total phenolic compounds was measured by using a linear calibration curve (R^2^ > 0.995) of gallic acid (Sigma, USA) solution. The results were expressed as milligrams of gallic acid equivalents (GAE) per liter of wine (mg GAE/L). All measurements were performed in duplicate.

*Antioxidant activity—*The total antioxidant activity of wines was measured using the ABTS radical cation decolorization assay, according to Re et al. [46]. ABTS (Sigma, USA) was dissolved in methanol to a concentration of 7 mM. The ABTS radical cation was produced by reacting ABTS stock solution with 140 mM potassium persulfate (Sigma, USA) and allowing the mixture to stand in the dark at room temperature for 12–16 h before use. The ABTS radical cation solution was diluted with ethanol to obtain the absorbance of 0.70 ± 0.02 at 734 nm. An aliquot of each wine prepared in three different dilutions (10 µL) was mixed with 990 mL of diluted ABTS radical solution. After reaction at room temperature for 15 min, the reduction in absorbance was measured at 734 nm. The reducing capacity of experimental wines was calculated with reference to the Trolox calibration curve (R^2^ > 0.997). The Trolox calibration curve, obtained using Trolox solution (Sigma, USA) with a concentration ranging from 0.5 to 2.5 mM in ethanol, was plotted as a function of the decrease in absorbance (ΔA = Ablank − Asample) of ABTS radical scavenging activity. The antioxidant activity of the samples was calculated by TEAC (Trolox equivalent antioxidant capacity) assay and it was expressed as mM Trolox Equivalent (TE) per liter of wine (mM TE/L). All solutions were prepared daily. All measurements were performed in duplicate.

### 2.4. Aromatic Compounds

Concentrations of main secondary compounds, such as acetaldehyde, ethyl acetate, acetoin and higher alcohols, were determined by direct injection of wine samples into a glass column packed with 80/120 Carbopak B/5% Carbowax 20 M (Supelco, Sigma-Aldrich, Milano, Italy) by an Agilent 7890A gas-chromatograph, as reported by Capece et al. [47]. The experimental wines produced at pilot scale were analyzed also for the content in esters, alcohols, aldehydes and terpenes by headspace solid-phase-microextraction sampling (SPME), using poly (dimethylsiloxane) (100PDMS) fiber (Supelco, Sigma-Aldrich, Milano, Italy) and GC-MS. A DB-WAXTER (Agilent) column (length 30 m, i.d. 0.250 mm) was used as reported by López-Martínez et al. [48]. The analysis was performed in splitless mode and the following conditions were used: 220 °C as injection temperature, 250 °C as detector temperature, helium as carrier gas with a flow rate of 20 mL/min. The initial temperature was 40 °C and then it was raised to 240 °C at 7 °C/min. Ten mL of the experimental wines were placed in 20-mL vials and 2 g of NaCl and 100 µL of 1%(*v*/*v*) isooctane (internal standard) in ethanol (Sigma-Aldrich, Milano, Italy) were added.

### 2.5. Statistical Analysis

All the experimental data were analyzed by one-way ANOVA and Levene’s test was applied to verify the homogeneity of variance.

Tukey’s test was used to evaluate the differences in the main oenological characteristics and in the secondary compound levels among the mixed and the pure cultures in the wines obtained by laboratory scale fermentation. Furthermore, the data of the wines performed at pilot scale with and without SO_2_ addition were statistically analyzed by ANOVA. The results were considered significant at *p value* ≤ 0.05. Each test was carried out in duplicate, and the results were expressed as mean value with the associated standard deviation (±SD). The PAST software ver. 1.90 [49] was used for all the statistical analyses.

## 3. Results

### 3.1. Fermentation Trials at Laboratory Scale

The four selected *S. cerevisiae* strains used in this work were previously isolated from the spontaneous fermentation of grapes of different origin and were selected for some technological characteristics, such as fermentative vigor, hydrogen sulfide production and killer character (Table 1). All the strains resulted in being low producers of hydrogen sulfide, a desirable trait for wine starter cultures, considering the negative influence of high level of this compound on wine organoleptic quality. Furthermore, the strains were selected on the basis of characters potentially able to confer a competitive advantage toward other yeasts present in grape must, such as killer phenomenon and fermentative vigor. We selected ND and M1, as these strains were killer, whereas the other two strains resulted neutral, which means that, although unable to produce killer toxins, these strains resulted resistant to killer proteins produced by reference killer strains. All the strains were characterized by high fermentative vigor [50], with values ranging between 2 and 2.5 g of CO_2_ per 100 mL of grape must (Table 1), a trait directly correlated with speed of fermentation. On the basis of these characteristics, these strains are expected to be better adapted to conditions in non-sterile fermentation media, without sulfur dioxide addition. The four selected *S. cerevisiae* strains were tested at laboratory scale fermentations as mixed cultures, in which they were inoculated in co-cultures one with the other in different combinations (Figure 1), in comparison to single starter of each strain. All the fermentations, both inoculated with single strains and mixed starters, were completed successfully after 14 days, with a sugar residual less than 5 g/L.

As regards the fermentative vigor exhibited by the 10 starter cultures, single and mixed starters exhibited similar fermentative vigor, with values ranging between 18.2 and 24.8 g of CO_2_ per 500 mL of grape must (Figure S1), with the lowest value showed by a M1 single starter, whereas the highest fermentative vigor was found for ND-SA mixed starter.

#### 3.1.1. Strain Dominance Ability during Lab-Scale Fermentation

**Figure 2:**
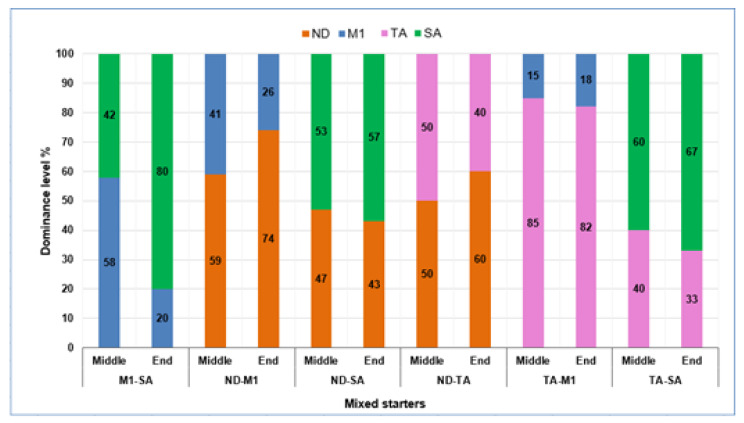
Dominance percentage of strains included in mixed starters during laboratory-scale fermentation in the middle and final phases of the process. For each mixed fermentation and process phase (middle and end), the dominance percentage of each of the two strains composing the mixed starter is reported (i.e., for M1-SA mixed fermentation, the dominance percentage of M1 and SA strains is reported). Middle = fermentation stage with the reduction of 50% of the sugars. Data are mean of two independent experiments.

At different stages of the fermentations, in particular at middle (50% of sugar consumption) and at the end of the fermentative process, the dominance ability of inoculated starters was evaluated by isolation on WL medium. For each sample and fermentation time, thirty isolates, randomly chosen, were identified by restriction analysis of the 5.8 S-ITS region as *S. cerevisiae* species and were characterized by amplification of the interdelta region with the primer pair δ2/δ12. The comparison between the molecular profiles of the inoculated strain/strains (for single and mixed fermentations, respectively) and molecular patterns of yeasts isolated during fermentations allowed to evaluate the starter dominance ability (for single fermentation), and, in the case of mixed fermentations, the percentage of dominance of each strain included in the mixed cultures. In the case of single starter fermentations, the dominance ability of starter was 100% for all the trials. As regards the six mixed starter fermentations, the dominance rate of each strain included in the mixed culture at the different sampling times, expressed as a percentage of colonies showing the same molecular profiles of the two strains composing the mixed starters, is reported in Figure 2. In both the fermentation phases, indigenous/unknown strains were not found. In the middle stage of fermentation, three different results were found:

- in three mixed fermentation (M1-SA, ND-M1, TA-SA), a slightly higher prevalence of one strain than the other was found (a ratio of about 40% and 60%);
- in two mixed fermentations (ND-SA and ND-TA), the two strains were present, with a percentage very similar between them;
- in one case (TA-M1), a very high prevalence of one strain was found, in particular the strain TA was found with a frequency of about 80%, whereas M1 strain was present at a very low percentage (about 20%).

At the end of the fermentative process (Figure 2), different results were found. In general, the highest percentage of dominance was exhibited by SA, dominating in M1-SA and TA-SA mixed fermentations, and ND, which resulted predominant in ND-M1 and ND-TA mixed fermentations.

Both these two strains might be considered to be characterized by high dominance ability; in fact, they showed a very similar level of dominance when they were used together as a mixed starter (ND-SA). Otherwise, M1 strain might be classified as strain possessing a very low dominance ability; in fact, in all the mixed trials including M1, its percentage of dominance ranged between 18% (TA-M1) and 26% (ND-M1).

#### 3.1.2. Analysis of Experimental Wines at Laboratory Scale

Table 2 lists the values for chemical parameters, such as free SO_2_ content (at middle and end phase), polyphenols and antioxidant activity, and level of main secondary compounds influencing wine aroma, found in the experimental wines obtained by single and mixed starters. For each wine from mixed starters, the data were compared by one-way ANOVA with data found in the two wines obtained by the corresponding single starters (i.e., data detected in wine from M1-SA starter were compared with values detected in wines obtained with M1 and SA single starters).

Generally, the highest differences between single and mixed starter wines were found for ND-SA and ND-TA, whereas the lowest differences were found for M1-SA.

**Figure 3:**
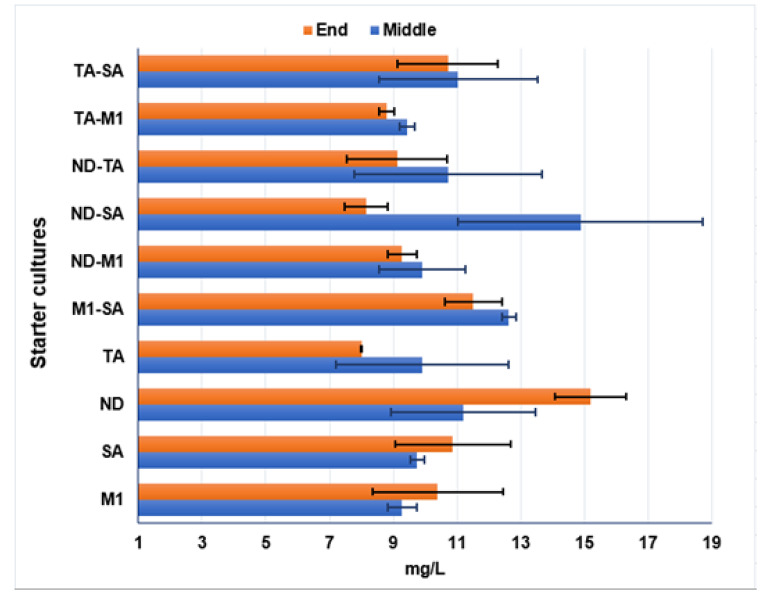
Free SO_2_ content (mg/L) in the samples obtained by single and mixed starters determined at middle and end phase of the fermentation process. See Table 2 for the results obtained by ANOVA analysis.

*Determination of SO_2_* — As regards free SO_2_ (Table 2, Figure 3), a reduction of content during the fermentation was observed in all the wines obtained by mixed cultures; in fact, the free SO_2_ level at the end of the fermentation was lower than the amount detected in the middle step of the process. Contrarily, an increase of free SO_2_ at the end of the process was observed in wines from single starters and this increase was quite high for wine obtained by inoculating ND, with 11.20 mg/L detected at the middle step of fermentation, whereas a level of 15.20 mg/L was found in the final wine (the highest value of free SO_2_ content found among all the experimental wines analyzed in this step). A reduction of free SO_2_ during the process was observed only in wines obtained by inoculating TA strain (values of 9.92 and 8.00 mg/L in the middle and final step of fermentation, respectively). The level found in the final wine obtained by inoculating TA strain was the lowest value of free SO_2_ detected in this step. As regards the comparison between mixed and single starters wines, no significant differences were detected for free SO_2_ levels in the middle step of fermentations for all the samples. At the end of the fermentation process, significant differences (*p* ≤ 0.05) were found for free SO_2_ content in the wines obtained by ND-M1, ND-SA and ND-TA, compared to the corresponding single strains wines.

*Determination of total polyphenols content and antioxidant activity of experimental wines*—The experimental wines were analyzed for the content of total polyphenols and antioxidant activity, in order to evaluate the influence of starter cultures on these parameters. With regards to the polyphenol content, a certain variability was observed among the experimental wines, although no significant differences were found for this parameter between wines from mixed starters and the corresponding single starter fermentations. The number of total polyphenols ranged from 108.74 mg GAE/L, found in wine obtained with mixed starter ND-TA, to 200.82 mg GAE/L, detected in wine from ND single starter (Table 2).

The TEAC (Trolox equivalent antioxidant capacity) assay was used to measure the total antioxidant activity of the experimental wines and the results were expressed as mM Trolox Equivalent (TE)/L. The highest level of TEAC was found in wines from mixed starters ND-SA, ND-M1 and ND-TA, whereas the lowest values of this parameter were detected in wines obtained by single starters M1 and mixed cultures M1-SA and TA-SA. For ND-SA and ND-TA, no significant differences were found between wines obtained with mixed starter cultures and the corresponding single starter wines, as both the single strains composing the mixed starter yielded wines characterized by high values of TEAC. Conversely, for wines obtained by inoculating ND-M1 and TA-M1, statistically significant differences between wines from mixed starters and the corresponding single starter wines were found, as M1 starter produced wine characterized by lower TEAC (2.89 mM TE/L wine) than the level detected in wines produced by ND and TA single starters (4.71 and 4.76 mM TE/L wine, respectively). Significant differences were also found for the wines produced by TA-SA in comparison to its single strains; in this case, the mixed starter wine showed the lower TEAC value (2.43 mM TE/L wine) than the amount determined in wines produced by TA and SA single starters (4.76 and 4.09 mM TE/L wine, respectively).

*Volatile acidity*—In all the samples, this parameter was included in the acceptable level, with the maximum level detected in wine obtained by TA single starter (456 mg/L). Furthermore, all the wines obtained with mixed starters contained a level of volatile acidity statistically different from the levels found in the corresponding single starter wines, except for M1-SA (Table 2).

*Aromatic compounds*—Table 2 also shows the average and standard deviations of some volatile compounds detected in the different fermentations, comparing each mixed fermentation with the corresponding single starter fermentations. With regards to the acetaldehyde content, in all the wines the level was included in the usual concentration reported (from 10 to 75 mg/L), except for the wine obtained by M1 single starter. In fact, in wine from M1 strain, an acetaldehyde level of 112 mg/L was found, although in wines obtained by M1 in association with other strains, this compound was found at low level. In particular, in M1-SA wine the acetaldehyde amount was 38.66 mg/L; in TA-M1, wine was 37.53 mg/L and 46.14 mg/L in wine obtained by ND-M1. For this compound, statistically significant differences between mixed starter wines and the corresponding single starter wines were found in almost all the samples, except for ND-TA. Ethyl acetate content ranged between 9.08 and 12.61 mg/L and statistically significant differences were found only for ND-TA and TA-M1.

Additionally, acetoin, aliphatic alcohols, such as *n*-propanol, isoamyl alcohol, isobutanol and active amyl alcohol were present at the concentrations usually reported in wine [51]. As regards acetoin, significant differences were found in all the wines obtained from mixed starters in comparison to the wines fermented with the corresponding single starters, except for wine obtained by ND-TA mixed starter. For *n*-propanol and isobutanol levels, differences were found only in wines from two mixed fermentations (ND-SA and ND-TA, TA-SA and ND-TA, respectively) with the corresponding single starter wines, while levels of amyl alcohols, in particular of isoamyl alcohol, detected in all the wines from mixed starters, were statistically different from the amounts found in the wines fermented with the corresponding single starters.

### 3.2. Pilot Scale Vinifications

On the basis of the results obtained by laboratory scale fermentations, the mixed culture ND-SA was chosen and tested as starter at pilot scale vinification, in a winery producing “Aglianico del Vulture” wine. The criteria used for selecting this starter were based on the characteristics of wine obtained by the laboratory scale fermentation with ND-SA, that is low free SO_2_ content (8.16 mg/L), high antioxidant activity (5.55 mM TE/L) and a desirable level of higher alcohols (<300 mg/L). Furthermore, the very similar percentage of dominance of both the strains composing the mixed starter during the fermentation might indicate the contribution of both the strains to the process and, potentially, to final quality of the wine. The mixed culture was tested in two conditions, with and without SO_2_ addition, by using a higher inoculum level for the vinification without SO_2_.

#### 3.2.1. Mixed Starter Dominance during Pilot Scale Vinifications

The dominance of starter culture was evaluated at middle and final step of the fermentation by analyzing the yeast colonies isolated. Non-*Saccharomyces* yeasts were found in both the fermentation trials and phases analyzed (data not shown). In wine with SO_2_ addition we found a non-*Saccharomyces* population of 1.50 × 10^7^ CFU/mL and 2.50 × 10^6^ CFU/mL at middle and end fermentation phases respectively, while in wine without SO_2_ addition this population was 1.70 × 10^7^ CFU/mL in middle stage and 5.90 × 10^6^ CFU/mL at the end of the fermentative process.

The isolates belonging to *S. cerevisiae* species were submitted to inter-δ region analysis and the strain dominance was calculated on the basis of the percentage of colonies showing the same pattern of the two strains composing the mixed inoculated starter.

**Figure 4:**
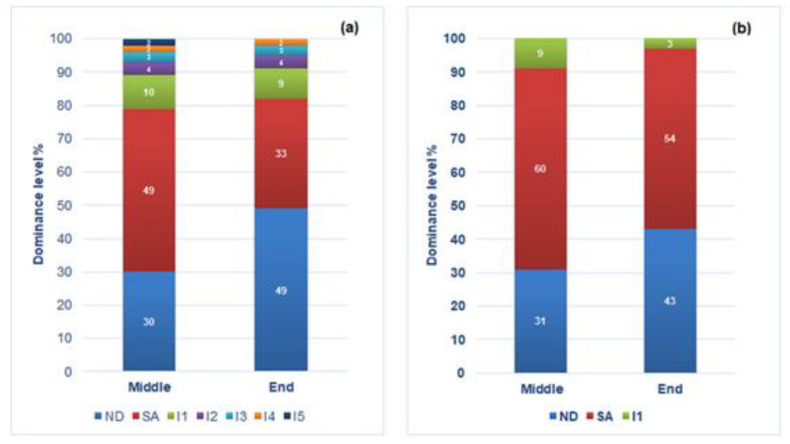
Dominance level of the mixed starter ND-SA used for the two pilot-scale vinifications, with (**a**) and without (**b**) SO_2_ addition in the middle (50% of the sugar reduction) and the final phase of the fermentation.

The results related to starter dominance obtained in the two fermentation tests were reported in Figure 4 as an average of the duplicate experiments. In the vinification carried out with the addition of SO_2_ (Figure 4a), at the middle stage of the process, the dominance ability of ND and SA strains was 30% and 49% respectively. *S. cerevisiae* different from the inoculated strains were found at a percentage of 21%, with the presence of 5 different molecular profiles coded as I1 (10%), I2 (4%), I3 (3%), I4 (2%) and I5 (2%). In this experiment, at the end of the process, the dominance ability of inoculated strains was 49% (ND) and 33% (SA). In this step, *S. cerevisiae* strains with molecular profiles different from those of inoculated strains were found at a total percentage of 18%, with the presence of the same molecular profiles found in the middle stage, except for the I5.

As regards the vinification trial performed without addition of SO_2_, the results were reported in Figure 4b. At the middle stage of the process, the ND and SA strain dominance ability was 31% and 60% respectively, and only one profile different from ND and SA patterns was found, coded with I1. At the end of the fermentation, the dominance ability of the two strains included in the starter was 43% (ND) and 54% (SA) and the profile I1 (already found in the middle of the fermentation) was the only one differing from the patterns of the inoculated strains. Among all the isolates showing molecular profiles different from interdelta patterns of ND and SA, the profile I1 was found among the yeasts isolated from both the experiments in both the phases of the processes.

This strain might be an indigenous strain coming from the grape must, or a strain resident in the cellar.

#### 3.2.2. Analysis of Wines Obtained at Pilot-Scale

##### Oenological Parameters

Table 3 reports means and standard deviation of the main oenological parameters determined at the end of fermentation in wines obtained at pilot scale vinifications performed with (+SO_2_) and without SO_2_ (-SO_2_) addition. With regards to ethanol, glucose and fructose residual, acidity (both total and volatile) and total SO_2_, no differences were found between the two conditions. Finally, statistically significant differences were found for the content of free sulfur dioxide, as expected, with level significantly lower for the wines obtained without SO_2_ addition. Additionally, the levels of total polyphenols (mg GAE/L) and antioxidant activity (mM TE/L) were significantly different between the wines produced with and without SO_2_ addition (Table 3). In particular, NO-SO_2_ added wine showed higher content of these compounds than in the other condition. With regards to the polyphenols content, the values ranged from 581.25 mg GAE/L in SO_2_ added wine to 721.15 mg GAE/L in NO-SO_2_ added and the level of TEAC was of 4.71 and 5.15 (mM TE/L wine) in the two types of wine (SO_2_ added and NO-SO_2_ added, respectively).

##### Content of Main Secondary Compounds

**Figure 5:**
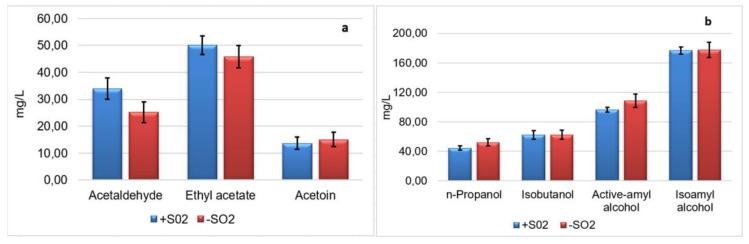
Main volatile compounds (**a**,**b**) produced during pilot-scale vinifications by mixed starter (ND-SA) in two trials carried out with (+SO_2_) and without SO_2_ addition (-SO_2_). Data are expressed in mg/L and are mean ± SD of two independent experiments.

The content of volatile compounds at the highest concentration in wine, such as acetaldehyde, ethyl acetate, and some higher alcohols (*n*-propanol, isobutanol and amyl alcohols) is reported in Figure 5a,b. Only acetaldehyde content differed in function of the treatment (with or without SO_2_ addition), with levels significantly lower in wine obtained without SO_2_ addition, in particular 33.93 mg/L in wine produced with SO_2_ and 25.19 mg/L in wine without sulfite addition.

Ethyl acetate is the most important ester in wine, playing a positive influence on wine aroma in a range of 50–100 mg/L [52]. No difference was found for this compound in the two types of wines (SO_2_ added and NO-SO_2_ added) at the end of the fermentative process (50.09 and 45.82 mg/L, respectively).

Acetoin is a key compound produced by yeast metabolism, in particular by non-*Saccharomyces* yeasts activity, contributing to wine aroma as a precursor of other aromatic molecules. Generally, in wine, the acetoin content ranges between 2 to 32 mg/L. In our study, at the end of the fermentative process, the acetoin level was 13.70 mg/L in SO_2_ added wine and 15.04 in NO-SO_2_ added wine (Figure 5a). No influence of SO_2_ on the content of this compound was found, whereas the highest level found in the wines just after fermentation might be correlated to the metabolic activity of non-*Saccharomyces* yeasts, present in both the middle and final phases of the fermentation.

With regards to the higher alcohols (Figure 5b), the content detected in all the experimental wines was included in the desirable level, contributing to the fruity aroma of the wines. For these secondary compounds, no differences were found between wines obtained with the two treatments.

##### Content of Volatile Compounds by SPME

The experimental wines were also analyzed for volatile compounds present in small amounts by SPME technique. A total of thirty-eight volatile compounds were identified, belonging to four chemical classes, esters (22 compounds), alcohols (8), terpenes (5), and aldehydes (3); compounds that, also in small amounts, can significantly affect the aromatic quality of wine. The results are summarized in Table 4, where the data are expressed as means ± standard deviation of two independent experiments for each vinification protocol. The data reported in this table were submitted to one-way analysis in order to evaluate the influence of the fermentation protocol (with and without SO_2_ addition) on the volatile composition. No significant differences were registered (*p* ≤ 0.05) for the majority of the compounds, except for some esters, such as ethyl propanoate, ethyl 2-methylbutanoate, ethyl 2-methylpropanoate, butyl acetate and isoamyl butyrate, indicating that the presence of SO_2_ did not affect the content of the majority of compounds detected by SPME. Ester class was the most abundant group in all the samples, followed by alcohols, terpenes and aldehydes.

Among the esters, isoamyl acetate (banana aroma), ethyl isobutyrate (fruity aroma), ethyl propanoate (pineapple aroma) and ethyl hexanoate (fruity, floral aroma) were present in high concentrations.

The higher alcohols present in the highest concentrations were 2-phenyl-ethanol and 1-butanol, which, in combination, constituted up to 67% of total alcohols, independently of the treatments applied. Other compounds analyzed were terpenes, which are mainly correlated to flowery aroma in wine. As shown in Table 4, four terpenes were detected in the experimental wines. The most represented was α-terpineol, characterized by a flowery aroma, which was detected at a value of 13.35 and 12.24 mcg/L in SO_2_ and NO-SO_2_ added wines, respectively, and β-damascenone, which has been reported to possess an apple flavor. With regards to aldehydes, the most represented was furfural, with values ranging from 102.64 to 93.36 mcg/L in SO_2_ and NO-SO_2_ added wines, respectively.

## 4. Discussion

Numerous chemical/physical approaches were proposed as potential alternatives to the use of sulfur dioxide in winemaking, whereas very few papers report scientific results on the possibility of the use of biotechnologies for reducing the addition of sulfur dioxide during winemaking [59].

Our study was addressed to propose a biotechnological approach, based on the use of a selected starter culture, able to perform wine fermentation without SO_2_ addition. In the first step of the research activity, four selected wild *S. cerevisiae* strains were tested as mixed starter cultures, in order to select the most suitable strain combination for fermentation without sulfur dioxide addition. The parameters used for choosing the four wild strains were mainly the fermentative vigor and killer character. In fact, the fermentation rate is one of the main characteristics assuring the dominance of *S. cerevisiae* strains in wine fermentation and preventing the development of undesirable microorganisms. Likewise, the killer character is one of the most studied biotic factors affecting the interaction and starter dominance among *S. cerevisiae* strains [27]. The strains used in the first step were killer or neutral strains, as killer strains are able to dominate over the natural microflora, inhibiting sensitive wild strains, whereas neutral strains are resistant to the action of killer toxins produced by killer wild strains. It was reported that the dominance of some strains during spontaneous fermentation is correlated to killer activity. The production of killer toxins confers a competitive advantage of these strains, excluding, from grape must fermentation, other yeasts present among the wild yeast population [47,60]. However, the precise mechanisms by which *S. cerevisiae* is able to inhibit other microorganisms during wine fermentation are still largely unknown. Indeed, the strain dominance during inoculated fermentation is not always directly correlated with killer activity. In our study, during mixed fermentation at laboratory scale with the four selected strains, the M1 strain (killer) showed the lowest percentage of dominance (Figure 2), both in mixed culture (ND-MI) with the killer ND strain and in mixed culture (TA-MI) with the neutral TA strain, with a dominance percentage of 18% and 26%, respectively. No correlation between starter dominance and killer activity was found also in other studies addressed to analyze the mechanisms of strain-to-strain interaction [61,62]. Perrone et al. [61] correlated the dominance character of some *S. cerevisiae* strains to cell-to-cell mechanisms, suggesting that the dominant behaviour of *S. cerevisiae* is only expressed when they sense other yeasts in the same environment. The M1 strain showing the lowest dominance ability in mixed starter fermentations was characterized by a very high production of acetaldehyde, when tested as a single starter (Table 2). This molecule was considered as a chemical compound involved in yeast “communication”. Different authors demonstrated that acetaldehyde is an interesting candidate as messenger molecule [63,64,65,66]; acetaldehyde exchange between strains may inhibit the growth of some yeast strains, while encouraging the growth of others. These mechanisms might play an important role in the interactions between yeasts composing mixed starter cultures [65]. All the mixed starters, including M1 strain (M1-SA, ND-M1 and TA-M1), were composed by strains with different acetaldehyde-producing level. The initial acetaldehyde level produced by M1 strain in the mixed starter fermentation may have favored the growth of the other *S. cerevisiae* strain included in the mixed starter (e.g., SA, ND and TA) that can function as receptor strains and utilize the acetaldehyde produced by M1. This might explain both the dominance of the other strains included in the mixed starter with M1, mainly at the end of the fermentation, and the lower level of acetaldehyde detected in wines produced by the mixed starter compared to wine from M1 single starter (Table 2).

These results emphasize the existence of microbial interaction between the two strains composing the mixed starter, and demonstrate that each strain included in co-culture gives its contribution, affecting wine composition. Furthermore, the influence of strain on wine characteristics is not directly correlated with dominance percentage. In fact, it was expected that the mixed fermentation TA-M1, characterized by a high percentage dominance of one strain, i.e., TA (Figure 2), might produce a wine with characteristics very similar to those of the single starter wine obtained by TA, that is wine obtained by TA-M1 starter had to be very similar to wine from TA (Table 2). However, from our data, this similarity was found only for one among 13 wine parameters analyzed (acetaldehyde, Table 2).

In consequence of these results, it was decided to test if the synergistic interactions between strains in mixed cultures can give an advantage to mixed cultures, rather than single starter, toward the natural microflora of grape must, in order to use this starter in vinification without sulfur dioxide addition. On the basis of these considerations, the most promising starter culture, selected on the basis of the results obtained at laboratory scale, was tested at pilot scale vinification in a winery producing “Aglianico del Vulture”. Our aim was to propose a biotechnological tool, based on the use of a selected starter culture, together the management of fermentation through the use of higher doses of starter in vinification without sulfur dioxide addition. As already reported, only a few studies used a similar approach to test the suitability to produce wine without sulfite addition.

Comuzzo and Zironi [59] observed that the management of alcoholic fermentation by an early inoculation of active dry yeast, achieved by taking care in yeast rehydration and acclimatization in must, allowed to perform alcoholic fermentation without sulfite addition. In our study, the successful implantation of inoculated starter was evaluated by an analysis of starter dominance during the fermentation. At pilot scale, in vinification performed without SO_2_ addition the selected starter confirmed the high dominance ability showed in fermentation at laboratory scale, whereas lower dominance ability was found in the control trial, performed with sulfite addition and use of normal starter dosage. In winery vinification with SO_2_, *S. cerevisiae* strains different from inoculated starter were found at a percentage ranging between 21% and 18%, in the middle and end of fermentation, respectively. Furthermore, in trial with SO_2_-added it was found a number of strains different from starter higher than those found in vinification without SO_2_ addition (Figure 4). Only one strain, coded with I1, was common to both the trials; this strain was the only one found in the trial without SO_2_ addition and found with the highest percentage in the vinification with sulfite addition. Although it cannot be considered as a predominant strain as it was found with a percentage frequency lower than 30% [62], it might be an indigenous strain, coming from the grape must, or a strain resident in the cellar, and, probably, it possesses fitness conferring ability to adapt to the specific winemaking conditions used in that winery [67]. Our results demonstrate that the sulfite addition is not a guarantee to reach an absolute dominance of starter culture on indigenous microflora, whereas the high prevalence of inoculated starter was obtained by using a high inoculum dose of a selected starter culture. The analysis of experimental wines obtained in the two vinifications revealed no substantial changes in the chemical composition of the wines obtained at the end of the processes in function of the management of fermentations, except for polyphenol content and antioxidant activity (Table 3). In our study, the wine produced without SO_2_ addition was characterized by higher levels of these parameters than the SO_2_-added wine. Very few data are present in literature reporting the influence of sulfite addition on polyphenol content and antioxidant activity and the reported results are contradictory. Some authors [68] reported that polyphenolic compounds and antioxidant activity were higher in organic wines, compared to conventional wines, whereas other authors [4,69,70] reported not always significant differences between organic and conventional wines that were not always significant. In a very recent study performed in Aglianico red wine [71], for the first time, the biological effects of wines with different SO_2_ content (free, low and conventional amount) were evaluated on an *ex - vivo* model of human erythrocytes.

Although these researchers found higher concentrations of bioactive compounds in wines with higher levels of sulfur dioxide, their results also demonstrated comparable biological activities in both SO_2_-free and low-sulfite wines compared to conventional wines, concluding that free- or low-sulfites wines have a great nutraceutical potential, providing a good intake biological activity.

As regards the influence of winemaking treatment (use of SO_2_ and inoculum level) on aromatic quality of wine, among the main secondary compounds affecting wine aroma (Figure 5), only a reduction of acetaldehyde was found in wine obtained without sulfite addition (Figure 5a). These results are in agreement with previous experiments carried out at winery scale without any addition of sulfur dioxide [59]. It is well known [52] that the concentration of the acetaldehyde is affected by the SO_2_ content; in fact, these two parameters are correlated by linear regression analysis, and the presence of SO_2_ promotes the production of acetaldehyde by *S. cerevisiae* [72]. Our results confirm this trend, with the lowest amount of acetaldehyde in wines with the lowest SO_2_ content.

Moreover, the content of majority of volatile secondary metabolites detected by SPME was not affected by SO_2_ addition, as statistically significant differences between the wines produced during the two vinifications were found only for a few compounds. Different results are available regarding the influence of SO_2_ on wine aroma [73,74,75], but it should be noted that mainly these studies have compared data between SO_2_-added wines and wines with alternatives to SO_2_, expecting an influence on the organoleptic character of wine, since a SO_2_ replacement alters wine aroma composition.

Another study on the effects of SO_2_ on volatile composition was performed by Garde-Cerdán et al. [76], who found an influence of SO_2_ on alcohols, esters and acids detected in the wines, but the grape must used in this study was submitted to PEF (pulsed electric field).

Recently, some researchers, investigating the effect of only SO_2_ addition, although at different levels, found an influence of sulfite addition on wine aroma. However, in these studies different conditions were tested simultaneously, such as the use of different strains [77,78] or the evaluation of SO_2_ addition effects and vineyard pied de cuve inoculation [79]. In our study, conversely, the comparison was made between wines with and without SO_2_ addition, obtained by the same grape must and using the same starter culture, and it is well known that the production of secondary compounds is affected by different factors, such as the composition of grape must and yeast strains performing the fermentative process.

In conclusion, the results of this study can be used as preliminary study, useful to set up a biotechnology-based strategy for the production of wine with a reduced content of sulfur dioxide. On the basis of scientific results obtained up until now, a complete elimination of sulfiting is currently not feasible, but the use of specifically selected starter culture, together with the use of good quality grapes, i.e., in good sanitary state, and the correct management of fermentation, can be proposed as an alternative for the production of low-sulfite wines. This approach confirms the high potentiality of microbial indigenous resource for innovations in the wine sector.

The strategy proposed in this study can satisfy the increasing consumers’ interest toward the healthy aspects of food and beverages and the negative perception of the sulfites. In fact, the proposed biotechnological tool allowed to produce good quality wines possessing “functional properties”, as, other than to assure the reduction of the risk of adverse effects related to sulfites, these wines were characterized by high polyphenol content and antioxidant activity. 

## Figures and Tables

**Table 1:** Technological characteristics of the four *S. cerevisiae* strains used in this work.

Strain Code	Origin	FV *	H_2_S Production	Killer Character	References
RB3-7Sc2 (ND)	Nero d’Avola	2.15 ± 0.02	low	+	Capece et al. [38]
TA4-10 (TA)	Inzolia	2.05 ± 0.02	low	neutral	Capece et al. [39]
M1-47 (M1)	Aglianico	2.00 ± 0.01	low	+	Capece et al. [40]
SA7-13 (SA)	Sangiovese	2.50 ± 0.04	low	neutral	Romano et al. [41]

* = fermentative vigor, evaluated at the first two days of fermentation (g CO_2_ evolved by 100 mL of Aglianico grape must).

**Table 2:** Main oenological characteristics and secondary compound content (mg/L) of the experimental wines produced by the mixed cultures in comparison to those obtained by the pure cultures. For each parameter, superscript letters mean significant differences at *p* ≤ 0.05 among each mixed fermentation and its single starters, i.e., MI-SA in comparison to M1 (first) and SA (second) single strains. Data are expressed as average ± SD of two independent experiments.

Starters	Free SO_2_ Middle End		TP	AA	Volatile Acidity	Acetaldehyde	Ethyl Acetate	Acetoin	*n*-Propanol	Isobutanol	Active-amyl Alcohol	Isoamyl Alcohol
M1-SA	12.64 ± 0.23	11.52 ± 0.91	165.05 ± 8.59	2.83 ± 0.40	205.58 ± 18.25	38.66 ± 10.34 ^a^	10.15 ± 0.23	2.79 ± 0.85 ^a^	46.09 ± 9.38	37.20 ± 3.95	108.99 ± 12.76 ^b^	198.14 ± 11.11 ^b^
ND-M1	9.92 ± 1.36	9.28 ± 0.45 ^a^	167.26 ± 16.69	5.45 ± 0.68 ^b^	214.82 ± 16.83 ^a^	46.14 ± 10.83 ^b^	9.96 ± 1.54	6.21 ± 1.2	38.75 ± 6.48	34.94 ± 8.89	118.03 ± 29.13	218.55 ± 11.60
ND-SA	14.88 ± 3.85	8.16 ± 0.68 ^a^	134.14 ± 17.95	5.55 ± 0.53	215.26 ± 5.96 ^ab^	29.56 ± 0.84	10.17 ± 0.98	7.02 ± 0.20 ^ab^	43.13 ± 0.53 ^a^	23.90 ± 0.16	91.25 ± 8.856 ^ab^	155.38 ± 2.43 ^ab^
ND-TA	10.72 ± 2.94	9.12 ± 1.58 ^a^	108.74 ± 0.78	4.83 ± 0.17	248.45 ± 8.09 ^ab^	32.28 ± 6,55	9.08 ± 0.80 ^b^	4.20 ± 1.19	43.72 ± 0.97 ^ab^	21.94 ± 1.21 ^ab^	101.51 ± 3.50 ^ab^	177.77 ± 4.30 ^ab^
TA-M1	9.44 ± 0.23	8.80 ± 0.23	137.45 ± 7.03	3.85 ± 0.05 ^a^	243.96 ± 12.53 ^ab^	37.53 ± 1.79 ^b^	10.04 ± 0.59 ^a^	9.86 ± 0.60 ^a^	34.61 ± 2.03	30.36 ± 0.96	99.27 ± 7.69	179.35 ± 5.10 ^a^
TA-SA	11.04 ± 2.49	10.72 ± 1.58	153.46 ± 18.10	2.43 ± 0.07 ^ab^	272.56 ± 24.55 ^ab^	32.73 ± 2.74 ^a^	10.35 ± 0.25	7.39 ± 0.83 ^b^	49.63 ± 4.96	27.62 ± 2.77 ^a^	94.66 ± 6.75 ^ab^	169.24 ± 15.74 ^ab^
M1	9.28 ± 0.45	10.40 ± 2.04	147.38 ± 17.95	2.89 ± 0.70	179.48 ± 16.36	112.92 ± 9.75	10.07 ± 0,20	9.34 ± 0.26	63.01 ± 13,79	45.66 ±7.40	109.84 ± 7.28	174.84 ± 17.16
ND	11.20 ± 2.26	15.20 ± 1.13	200.82 ± 13.53	4.71 ± 0.15	392.55 ± 4.36	44.04± 6.55	11.68 ± 0.51	3.54 ± 1.12	54.72 ± 1.16	29.39 ± 0.80	126.47 ± 1.81	246.99 ± 4.56
SA	9.76 ± 0.23	10.88 ± 1.81	136.90 ± 12.49	4.09 ± 0.29	153.58 ± 7.75	26.78 ± 1.17	10.49 ± 0.81	1.84 ± 0.05	51.92 ± 4.41	28.48 ± 2.22	60.90 ± 10.57	116.41 ± 14.44
TA	9.92 ± 2.72	8.00 ± 0.01	136.34 ± 17.95	4.76 ± 0.21	456.87 ± 3.66	48.34 ± 3.41	12.61 ± 0.52	5.59 ±0.80	65.49 ± 0.78	39.18± 1.06	127.77± 4.30	277.69 ± 2.27

TP = Total polyphenols expressed as mg GAE/L; AA = Antioxidant activity expressed as mM TE/L. ^a^ = significant difference with the first strain of the mixed cultures; ^b^ = significant difference with the second strain of the mixed cultures; ^ab^ = significant difference with both strains of the mixed cultures.

**Table 3:** Main oenological parameters in wines obtained with the mixed starter ND-SA at pilot-scale vinifications with (+SO_2_) and without SO_2_ addition (-SO_2_). For each compound, superscript letters mean significant differences at *p* ≤ 0.05 among wines produced in the two different conditions. Data are expressed as mean value ± SD of two independent experiments.

*Parameters*	+SO_2_	-SO_2_
Ethanol *	13.35 ± 1.33	13.53 ± 1.41
Fructose **	4.30 ± 0.17	5.00 ± 0.43
Glucose **	0.40 ± 0.02	0.37 ± 0.02
Total acidity **	7.80 ± 0.22	8.00 ± 0.69
Volatile acidity **	0.40 ± 0.11	0.39 ± 0.08
Malic acid **	2.10 ± 0.08	2.30 ± 0.09
Lactic acid **	0.00 ± 0.00	0.03 ± 0.01
Total SO_2_ **	35.02 ± 1.36	26.80 ± 1.49
Free SO_2_ **	30.15 ± 2.77 ^a^	11.36 ± 1.38 ^b^
TP	581.25 ± 14.19 ^a^	721.15 ± 7.12 ^b^
AA	4.71 ± 0.61 ^a^	5.15 ± 0.17 ^b^

* = expressed as ***v*/*v*%;** ** = expressed as g/L; TP = total polyphenols, expressed as mg GAE/L; AA = antioxidant activity, expressed as mM TE/L.

**Table 4:** Data (mean ± SD) of volatile compounds (mcg/L) determined by SPME in wines obtained with the mixed starter culture ND-SA in two vinification protocols: grape must with (+SO_2_) and without SO_2_ addition (-SO_2_). Odor descriptors (ODE) described in the literature are reported [53,54,55,56,57,58]. One-way ANOVA analysis and Tukey test were applied. * Significant differences between two wines at *p* ≤ 0.05.

Volatile Compound	+SO_2_	-SO_2_	ODE
Ethyl propanoate	137.76 ± 1.72 *	117.25 ± 2.69	Etherial, fruity, winey, pineapple
Ethyl isobutyrate	202.99 ± 10.37	210.59 ± 14.29	Fruity-like
Ethyl butanoate	1.15 ± 0.20	1.43 ± 0.19	Herbaceous fruit, strawberry
Propyl acetate	48.33 ± 5.49	53.17 ± 7.00	Etherial, fruity, sweet, banana
Isobutyl acetate	90.93 ± 8.41	94.22 ± 6.92	Sweet fruit, banana
Ethyl butyrate	92.00 ± 6.89	100.26 ± 9.48	Acid fruit
Ethyl 2-methylbutanoate	5.42 ± 0.27 *	4.26 ± 0.09	Sweet fruit, strawberry
Ethyl 3-methylbutanoate	1.83 ± 0.13	1.56 ± 0.23	Blackberry, berry, anice
Ethyl 2-methylpropanoate	1.89 ± 0.03 *	1.36 ± 0.05	Fruity, strawberry, pineapple
Butyl acetate	1.16 ± 0.04 *	1.48 ± 0.08	Sweet, ripe banana
Isoamyl acetate	473.33 ± 14.15	481.44 ± 11.62	Banana, pear
Methyl hexanoate	1.54 ± 0.16	1.75 ± 0.23	Fresh, fruity, pineapple, sweet
Ethyl hexanoate	97.20 ± 8.65	106.35 ± 9.43	Green apple
Isoamyl butyrate	2.97 ± 0.27 *	2.33 ± 0.40	Fruity, green apricot, pear, banana
Hexyl acetate	6.27 ± 1.43	8.69 ± 2.21	Fruity, green, pear
Ethyl heptanoate	1.01 ± 0.09	1.07 ± 0.22	Fruit
Ethyl trans-2-hexenoate	1.27 ± 0.13	1.32 ± 0.18	Waxy
Ethyl octanoate	8.23 ± 1.75	9.43 ± 2.05	Sweet, soap, pineapple
Isoamyl hexanoate	4.04 ± 0.32	3.63 ± 0.42	Fruit, flower
Isoamyl octanoate	2.87 ± 0.40	2.74 ± 0.33	Fruity, flowery
Ethyl phenylacetate	7.44 ± 0.96	6.57 ± 1.35	Rose petal
2-Phenylethyl acetate	87.93 ± 11.79	80.45 ± 8.65	Pleasant, flowery, rose
1-Butanol	275.635 ± 23.91	288.06 ± 12.90	Fusel, spiritous
1-Pentanol	1.89 ± 0.25	2.23 ± 0.17	Balsamic
4-Methyl-1-pentanol	1.31 ± 0.11	0.91 ± 0.04	Nutty
2-Heptanol	0.77 ± 0.09	0.85 ± 0.06	Green grass
1-Hexanol	106.54 ± 8.94	96.67 ± 7.91	Green grass
cis-3-Hexen-1-ol	98.27 ± 10.10	109.32 ± 8.49	Green grass
Benzyl alcohol	140.44 ± 15.69	132.62 ± 9.07	Phenolic balsamic, bitter almond
2-Phenylethanol	425.75 ± 38.22	412.29 ± 12.25	Rose
Linalool	11.23 ± 1.47	12.5 ± 1.61	Rose
α-Terpineol	12.24 ± 3.37	13.35 ± 3.49	Thrush, flowery
β-Citronellol	10.09 ± 2.86	8.56 ± 1.24	Citronella
Geraniol	7.23 ± 0.64	6.54 ± 1.01	Rose-like
β-Damascenone	13.43 ± 5.82	11.73 ± 1.31	Apple, Cooked apple
Benzaldehyde	25.16 ± 5.10	22.92 ± 2.75	Bitter almond-like, herbaceous
Hexanal	3.73 ± 0.59	4.17 ± 0.83	Green, woody, vegetative, apple, citrus
Furfural	93.36 ± 9.03	102.64 ± 5.20	Sweet, bread

## Supplementary Materials

Supplementary Materials can be found at https://www.mdpi.com/2076-2607/8/5/738/s1.
